# Asymptomatic Diagnosis of Huanglongbing Disease Using Metalloporphyrin Functionalized Single-Walled Carbon Nanotubes Sensor Arrays

**DOI:** 10.3389/fchem.2020.00362

**Published:** 2020-05-12

**Authors:** Hui Wang, Pankaj Ramnani, Tung Pham, Claudia Chaves Villarreal, Xuejun Yu, Gang Liu, Ashok Mulchandani

**Affiliations:** ^1^Key Laboratory of Modern Precision Agriculture System Integration Research, Ministry of Education and Key Laboratory of Agricultural Information Acquisition Technology, Ministry of Agriculture China Agricultural University, Beijing, China; ^2^State Key Laboratory of Animal Nutrition, Institute of Animal Science, Chinese Academy of Agricultural Sciences, Beijing, China; ^3^Department of Chemical and Environmental Engineering and Materials Science and Engineering Program, University of California, Riverside, Riverside, CA, United States; ^4^Escuela de Ciencia e Ingeniería de Materiales, Centro de Investigación y Extensión de Materiales, Instituto Tecnológico de Costa Rica, Cartago, Costa Rica; ^5^Research Institute of Wood Industry, Chinese Academy of Forestry, Beijing, China

**Keywords:** citrus greening disease, carbon nanotube, metalloporphyrin, chemiresistor, volatile organic compounds, artificial neural networks (ANN), gas sensor

## Abstract

Porphyrins, with or without metal ions (MPs), have been explored and applied in optical and electrochemical sensor fields owing to their special physicochemical properties. The presence of four nitrogen atoms at the centers of porphyrins means that porphyrins chelate most metal ions, which changes the binding ability of MPs with gas molecules via non-specific binding. In this article, we report hybrid chemiresistor sensor arrays based on single-walled carbon nanotubes (SWNTs) non-covalently functionalized with six different MPs using the solvent casting technique. The characteristics of MP-SWNTs were investigated through various optical and electrochemical methods, including UV spectroscopy, Raman, atomic force microscopy, current-voltage (I-V), and field-effect transistor (FET) measurement. The proposed sensor arrays were employed to monitor the four VOCs (tetradecene, linalool, phenylacetaldehyde, and ethylhexanol) emitted by citrus trees infected with Huanglongbing (HLB), of which the contents changed dramatically at the asymptomatic stage. The sensitivity to VOCs could change significantly, exceeding the lower limits of the SWNT-based sensors. For qualitative and quantitative analysis of the four VOCs, the data collected by the sensor arrays were processed using different regression models including partial least squares (PLS) and an artificial neural network (ANN), which further offered a diagnostic basis for Huanglongbing disease at the asymptomatic stage.

**Graphical Abstract:**
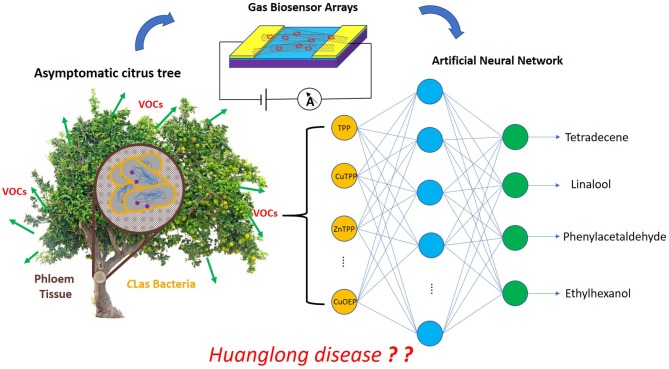
Huanglong disease diagnosed by hybrid chemiresistor sensor arrays.

## Introduction

Porphyrins (Auwarter et al., 2015; Gutiérrez-Cerón et al., 2016), known as one of the critical types of biological ligands, are macromolecular heterocyclic compounds consisting of four modified pyrrole rings, which are connected together through a methine bridge so that the molecule takes the form of a large ring (Zahou et al., 2016). Each pyrrole ring is made from four carbons and one nitrogen. All of the nitrogen atoms located at the interior of the large ring form a central cavity that can coordinate with most metal ions to form metalloporphyrin complexes (MPs). Due to the coordinated metal ions of MPs being in an unsaturated state, MPs can bind with one or two additional ligands at the axial position. MPs can combine with gas molecules through Van der Waal forces, hydrogen bonding, or interaction with the central metal ion (Penza et al., 2010; Shirsat et al., 2012). The optical and electrical properties will change sharply when MPs interact with gas molecules. Therefore, sensors decorated with different MPs or MP hybrid materials are considered to be excellent gas-sensing devices at room temperature (Liu et al., 2010), of which the sensing performance has been investigated by several groups. Song and coworkers (Song et al., 2016) used 5, 10, 15, 20-tetrakis(4-aminophenyl)porphyrin zinc (ZnTAP) and nanoporous anodized aluminum oxide membrane to assemble highly ordered nanotubes of ZnTAP, which was applied to detect NO_2_ at ambient temperature with high sensitivity and fast response-recovery time. Xie et al. developed a nitric oxide (NO) sensor based on a reduced graphene oxide field-effect transistor functionalized with iron–porphyrin. This gas sensor for real-time monitoring of NO released from cultured human cells measured NO in living cells with high sensitivity and specificity (Xie et al., 2016).

Single-walled carbon nanotubes (SWNTs) (Peng et al., 2016), one-dimensional structures with a nanometer-range diameter, are seamless cylinders comprised of a layer of graphene. They have gained widespread use in electrochemical sensors due to their interesting properties, such as excellent electrical conductivity, high surface-to-volume ratio, and thermostability (Salvetat et al., 1999; Kang et al., 2007). Studies indicate that gas molecules as either charge donor or a charge acceptor can be weakly adsorbed by SWNTs, which can cause a significant change in the electronic transport properties of SWNTs in view of charge transfer and charge fluctuation (Britz and Khlobystov, 2006; Giraldo et al., 2014). However, the low sensitivity and poor selectivity of bare SWNT-based sensors limit the ability of an individual sensor to analyze a multi-component gas mixture. Surface modifications that occur when SWNTs are functionalized with gas-sensitive materials can enhance sensing performance (Martin et al., 2013; Hijazi et al., 2014). MPs have flat and planar structures that efficiently bind with the SWNTs through π-π interactions (Bassiouk et al., 2013).

Huanglongbing (HLB), also called citrus greening or yellow shoot disease, is a bacterial disease of citrus caused by a vector-transmitted pathogen (Sagaram et al., 2009) that threatens the multi-billion dollar citrus industry all over the world (Bassanezi et al., 2011). There are two general means of propagation: (i) transmission from infected citrus trees to the surrounding trees by citrus psyllid insects (Grafton-Cardwell et al., 2013); (ii) grafting of an infected scion on a healthy citrus tree. In reality, if a citrus tree is infected by HLB, the disease will incubate for several years without any symptoms (Lee et al., 2015) and then exhibit visible symptoms, including yellowing of the veins, splotchy mottling of the entire leaf, dieback of twigs, and decay of feeder rootlets and lateral roots (Johnson et al., 2014). There are few effective and cheap treatments to cure this disease. The most frequently used method is to remove infected trees selectively, halting the spread of HLB, wherefore early detection of this disease is vital. In 2020, an approach to eradicate the bacteria responsible for Huanglongbing disease using silver nanoparticles (AgNPs) achieved remarkable results, providing a new method to cure this disease (Stephano-Hornedo et al., 2020). However, it is especially challenging to diagnose HLB at an early stage due to the infected trees lacking symptoms while still acting as reservoirs to transmit the disease. Current detection methods for HLB are based on qualitative assessment of disease symptoms and molecular analysis methods such as visible-near infrared spectroscopy (Sankaran et al., 2011), mid-infrared (MIR) spectroscopy (Sankaran et al., 2010), enzyme-linked immunosorbent assay (ELISA) (Rapala et al., 2002), and polymerase chain reaction (PCR) (Ananthakrishnan et al., 2010). These techniques are susceptible to error and require expensive, time-consuming processes, which makes them unsuitable for rapid, on-site detection at the asymptomatic stage. Previous research has demonstrated that the VOCs released by the trees are closely associated with plant metabolism, which offers a new means for detecting the status of plant health at any stage. Aksenov et al. (2014) used gas chromatography/differential mobility spectrometry (GC/DMS) to analyze the VOCs released by the Huanglongbing infected citrus tree. Hundreds of independent VOC measurements were collected and analyzed through GC/DMS. Based on changes in the concentrations of characteristic VOCs, the infection process was divided into four stages: healthy, asymptomatic, mild, and severe. They found that the contents of VOCs (tetradecene, linalool, nonadecane, phenylacetaldehyde, and ethylhexanol) changed dramatically at the asymptomatic stage. The levels of tetradecene and ethylhexanol deceased, and the rest increased. They established VOC-based disease detection with high accuracy. But GC/DMS is an expensive device that requires professional operation. To overcome this limitation, this paper proposes an electronic gas sensor to replace GC/DMS so as to determine the VOC concentrations at high sensitivity and low cost.

Here, hybrid chemiresistive sensor arrays based on SWNTs non-covalently functionalized with MPs was fabricated that is easy to use and low-cost and which is suitable for the detection of VOCs released from Huanglongbing infected citrus trees at the asymptomatic stage. The characteristics of MP-SWNTs were measured by optical and electrical methods, including UV-vis absorption spectroscopy, Raman, atomic force microscopy, current-voltage (I-V), and field-effect transistor (FET) measurement. The sensor arrays were applied to monitor the concentrations of the VOCs tetradecene, linalool, phenylacetaldehyde, and ethylhexanol. To discriminate the four VOCs qualitatively and quantitatively, partial least squares (PLS) and an artificial neural network (ANN) were further selected to process the data recorded by the sensor arrays.

## Materials and Methods

### Chemicals and Materials

Dispersed single-walled carbon nanotube solution (0.01 mg/ml, 95% semiconducting) was obtained from Nano-Integris Inc. (USA). Chemical reagents (dimethylformamide, acetone, propanol, and ammonium hydroxide) were purchased from Fisher Scientific Company (USA). Three volatile organic compounds (2-Ethyl-1-hexanol, 1-tetradecene, and phenylacetaldehyde) and 3-aminopropyltriethoxysilane (APTES) were purchased from Sigma Aldrich (USA). The MPs, namely tetraphenyl porphyrin (TPP), iron porphyrin (FeTPP), copper porphyrin (CuTPP), zinc porphyrin (ZnTPP), copper octamethyl porphyrin (CuOEP), and manganese OEP (MnOEP), were provided by two chemical companies, Sigma-Aldrich (USA) and Frontier Scientific (USA). Deionized water was used throughout the experiments.

The dispersed solutions of MPs (TPP, FeTPP, ZnTPP, and MnOEP) were prepared by dissolving a certain weight into 10 ml N, N-dimethylformamide (DMF) under ultrasonication. Saturated solutions of CuTPP and CuOEP were prepared for further use.

### Apparatus

The spectral, morphological, and electrical characteristics of SWNTs before and after being functionalized by MPs were investigated by atomic force microscopy (AFM), Raman, UV-Vis spectrometry, current-voltage (I-V), and field-effect transistor (FET) measurement. AFM images were obtained using an atomic force microscope (Veeco Innova, Santa Barbara, CA, USA). Raman spectra were measured with a Nicolet Almega XR Dispersive microscope with 532-nm laser excitation. The UV spectrum was acquired by a Beckman DU640 UV/Vis spectrophotometer (Beckman Coulter, Inc. USA). Electrical measurements were made using a semiconductor parameter analyzer (Keithley 2636, USA).

For FET measurements, the Si substrate was covered with gold film, which served as the base, and charged with a linear voltage ranging from −60 V to +20 V. The two gold electrodes etched on the SiO_2_ surface acted as the drain and source, to which a constant voltage (0.1 V) was applied. A dielectric layer of 100-nm thick SiO_2_ was used to separate the base from the source-drain.

### Fabrication of MPs-SWNTs

The sensor was designed with a single-gap structure because this offers a large area over which to react with gas molecules, and electrical conductivity can be controlled easily in comparison with an interdigital electrode. Highly p-doped silicon with a 100-nm SiO_2_ layer was employed to fabricate the single gap microelectrode (10 μm in width and 10 μm in length) by photolithography using the positive photoresist AZ-5214. A 20-nm Cr layer and a 180-nm Au layer were uniformly deposited and etched on the surface of the Si/SiO_2_ via e-beam evaporation. Finally, the photoresist residual was cleaned away with acetone solution.

**Figure 1:**
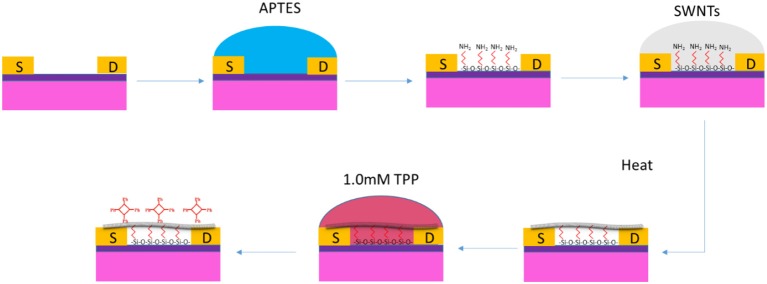
Schematics of preparation and operation of SWNTs functionalized by MPs.

The modification was shown in Figure 1. The single-gap electrode was flushed successively with acetone and isopropanol and then blow-dried in streaming air to remove surface impurities before use. The electrode was immersed into ammonium hydroxide for 30 min, and the residue was rinsed off with sufficient deionized water. The cleaned electrode was incubated in 0.5 mL APTES for 60 min, washed with deionized water, and blow-dried using a nitrogen stream as quickly as possible. After that, a 5-μL SWNT solution was added to cover the microelectrode, and it was incubated with a high-humidity and dark condition for 60 min. The residual SWNTs were then carefully cleaned away with deionized water, and the electrode was annealed in ambient air at 250°C for 60 min. For MP immobilization, the single-gap electrode decorated with SWNTs (SWNTs-FET) was immersed in different MPs and placed in the dark for 4 h. Finally, the MP-SWNTs was annealed at 90°C under inert gasses in a protected condition for 60 min.

### Gas Sensing Setup

The gas sensing setup shown in Figure S1 was designed and integrated to perform VOC determination. This device can generate different concentrations of VOCs by mixing different proportions of air and saturated vapors of VOCs, which flowed through a 1.2 cm^3^ sealed glass dome covering the sensor array. A Keithley 2636 was used as a data-collecting device, connecting to the sensor arrays via three meters (bias, drain, and source) and recording output voltage and input current. All devices were controlled by the software installed in the computer that was programmed with the Laboratory Virtual Instrument Engineering Workbench (Lab VIEW). For experimental measurement, a voltage of +0.1 V was applied between the drain and the source without the base voltage. Before detecting a desired concentration of VOC, the sensor array was exposed to dry air until it reached a stable baseline.

## Results and Discussion

### Characteristics of MP-SWNTs

Electrical characteristics (I_DS_-V_DS_ and I_DS_-V_G_), AFM, UV spectrometry, and Raman were used to investigate the characteristics of SWNTs before and after functionalization with MPs.

**Figure 2:**
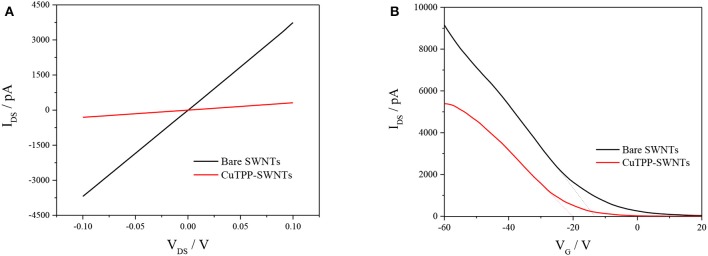
Electrical and FET transfer characteristics of a CuTPP-functionalized bare SWNT device: **(A)** I_DS_-V_DS_ at V_GS_ = 0 V and **(B)** V_G_-I_DS_ at V_DS_ = 0.1 V.

Electrical characterization is an effective method for distinguishing the changes in conductivity or resistance of SWNTs before and after each modification. As shown in Figure 2A, the I_DS_-V_DS_ curves of bare SWNTs and CuTPP-SWNTs presented good linear relationships, but the current decreased dramatically with CuTPP-SWNTs. As shown in Figure S2, the resistance values of bare SWNTs and CuTPP-SWNTs were 28 and 318 kΩ, respectively; thus, with the addition of CuTPP, the resistance increased 11 times in comparison with bare SWNTs. The poor conductivity of CuTPP-SWNTs illustrated that the molecules of CuTPP had interacted with the carbon nanotube sidewalls, forming specific π-π-interactions through non-covalent interactions (Li et al., 2004; Shirakawa et al., 2005). The change in conductivity may be attributed to a couple of different causes (Zhao and Stoddart, 2009): (i) the carrier concentration (n) may be changed by an electron/charge-transfer between CuTPP and SWNTs; (ii) CuTPP may act as a randomly distributed scattering potential, transforming the mobility (μ) of the charge carrier. The FET curves of bare SWNTs before and after functionalization with CuTPP are shown in Figure 2B, for which the two electrodes were exposed to the air. We found that the threshold gate voltage (V_TH_) of bare SWNTs was about −13.5 V, while when the SWNTs were functionalized with CuTPP, the FET curves shifted in the negative direction and the V_TH_ value was −20 V. According to the mobility equation, the mobility values of bare SWNT and CuTPP-SWNTs are 176 and 136 cm^2^/Vs, respectively, which is in agreement with the I_DS_-V_DS_ results. The results remarkably demonstrate that the positive hole of the p-type semiconductor SWNTs was occupied by the electron, resulting in a lower barrier concentration and carrier mobility.

**Figure 3:**
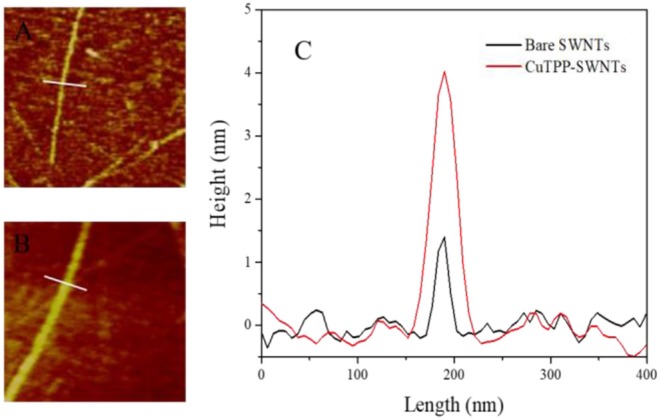
AFM images of **(A)** bare SWNTs and **(B)** CuTPP-SWNTs; **(C)** the height profile of bare SWNTs (Black) and CuTPP-SWNTs (Red).

The morphology of bare SWNTs and CuTPP-SWNTs was studied by AFM observation in Figure 3. It was clear that the height of bare SWNTs was about 1.7 nm, approximately equal to the theoretical value (Bandow et al., 1998), which also matched the diameter value for the SWNTs offered by the producer. The height of the CuTPP-functionalized SWNTs was noticeably increased to 4 nm as a consequence of the attachment of porphyrin to the SWNTs' surface.

**Figure 4:**
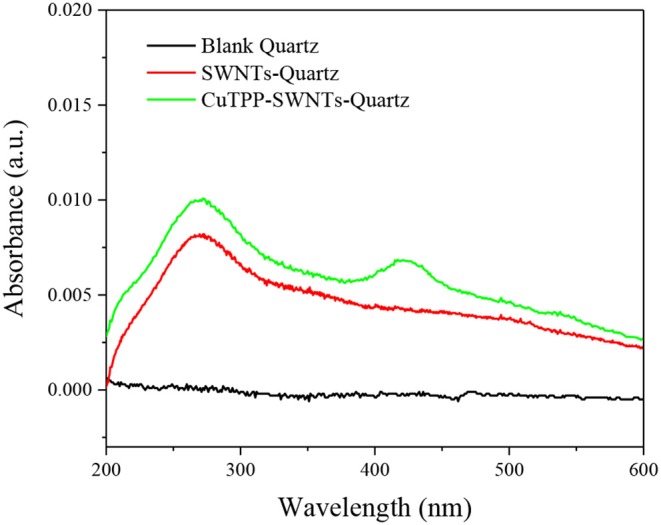
UV-vis spectra of blank Quartz (Black), SWNTs-Quartz (Red), and CuTPP-SWNTs-Quartz (Green).

The formation of CuTPP-SWNTs is further confirmed by UV absorbance because most MPs have a strong near-UV band [a sharp, intense Soret band (B band)] in the near UV region and two weak Q bands responsible for the red to purple color in the visible region. Here, we chose a quartz plate to replace the silicon substrate due to its good optical transparency. Compared to blank Quartz in Figure 4, SWNTs-Quartz had an absorption peak at 273 nm and an increasing absorbance intensity in the whole spectral range, suggesting that the opaque material of SWNTs had attached to the Quartz (Ryabenko et al., 2004). The network structure formed by this attachment hampers parts of light from moving through it. After SWNTs were functionalized with CuTPP, a transmitting B band at 418 nm and one Q band at 541 nm were observed, which demonstrate that CuTPP was covalently attached to the surface of the SWNTs (Mammana et al., 2008; Wang et al., 2011).

**Figure 5:**
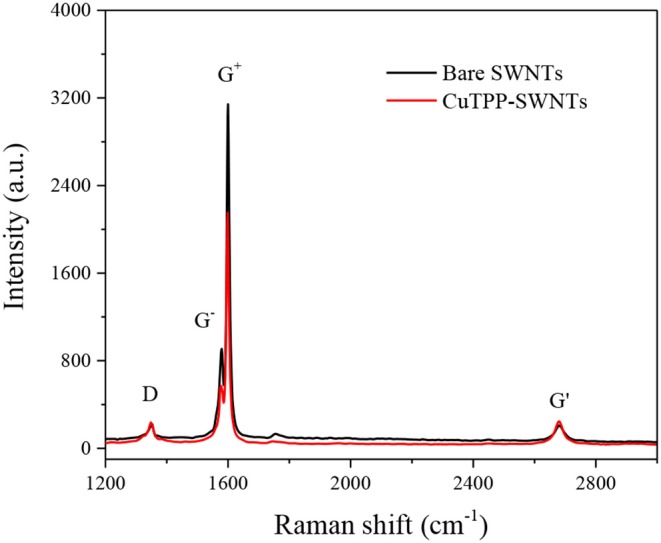
Raman spectra for bare SWNTs (Red) and SWNTs functionalization with CuTPP (Black).

The interactions between the SWNTs and CuTPP were characterized using Raman spectroscopy, and the results are shown in Figure 5. The Raman spectrum of SWNTs shows four peaks at 1,351 cm^−1^ (a small D band), 1,579 cm^−1^ (a G^−^ band), 1,600 cm^−1^ (a sharp G^+^ band), and 2,680 cm^−1^ (2D band). After CuTPP was non-covalently linked to SWNTs, the G^+^ band peak becomes narrower, and the G^+^ band shifts to 1,598 cm^−1^, suggesting that it can be ascribed to electron doping between SWNTs and CuTPP. Meanwhile, the D/G intensity ratio of CuTPP-SWNTs increase in comparison with SWNTs, indicating the conversion of sp2 carbons to sp3 carbons on the SWNTs surfaces due to the functionalization (Geng and Jung, 2010).

### Gas Sensing Performance

As discussed earlier, the VOCs can serve as biomarkers to diagnose whether a citrus tree is infected by HLB. To evaluate the sensing performance of MP-functionalized SWNTs devices, we measured the real-time electrical response of the hybrid chemiresistor sensor arrays to phenylacetaldehyde, tetradecene, linalool, and ethylhexanol vapors with concentrations varying from 5 to 100% of saturated vapors at room temperature. A normalization method was adopted to process the electrical signal, reducing the differences caused by the modification. The normalized response is defined as a relative change in resistance:

(1) $$\begin{array}{l} {\;}_{\Delta }\text{R}/{\text{R}}_{0}\;\%=\left(\text{R}-{\text{R}}_{0}\right)/{\text{R}}_{0}\times 100\% \end{array}$$

where *R*_0_ is the value of initial baseline resistance before MP-SWNTs are exposed to VOCs and *R* is the value of resistance after MP-SWNTs are exposed to VOCs.

**Figure 6:**
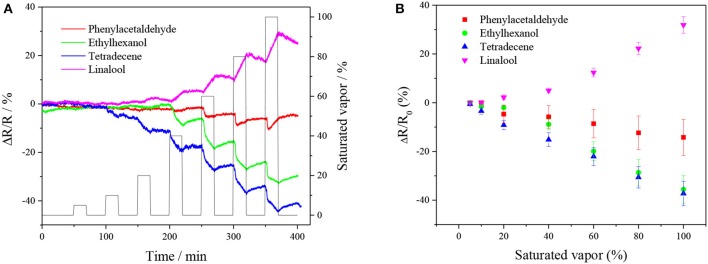
**(A)** The real-time relative responses and **(B)** calibration curves of CuTPP-SWNTs toward different concentrations of four VOCs varying from 5 to 100%.

Figure 6A shows the normalized resistance of CuTPP-SWNTs toward the four VOCs (tetradecene, linalool, phenylacetaldehyde, and ethylhexanol) released by infected citrus trees at the asymptomatic stage. The normalized resistance of CuTPP-SWNTs displayed a correlation with the VOC concentrations in the Figure 6B. The conductivity of CuTPP-SWNTs was increased after CuTPP-SWNTs had been exposed to tetradecene, ethylhexanol, and phenylacetaldehyde, while, in CuTPP-SWNTs exposed to linalool, the conductivity decreased. The reason for this is discussed in Mechanism section. Compared with the sensing responses of bare SWNTs shown in Figure S3, the sensitivity of CuTTP-SWNTs to the VOCs (except for phenylacetaldehyde) was improved to different degrees. For 100% saturated tetradecene vapor, the normalized response of bare SWNT is only 0.83%, with the response reaching 50% of the maximum in 4 min, as shown in Figure S4. However, the normalized response of CuTPP-SWNTs was 45 times higher than that of bare SWNTs at the same concentration, and 50% of the maximum response was reached in <5 min. Similar fast responses were observed using the rest of the MP functionalized SWNT devices to test the four VOCs; the related data are shown in Tables S1–S4. This indicates that the modification of SWNTs with MPs can enhance sensitivity, selectivity, and response time in detecting gas concentration. As shown in Table S5, the lowest detection limits were 4.86, 10.15, 0.18, and 0.61 ppm for phenylacetaldehyde, ethylhexanol, tetradecane, and linalool, respectively. After each experiment, we found that the sensor arrays needed a long time to recover to the baseline, but it was possible to shorten the recovery time by exposing the sensor arrays to UV light or a high-temperature environment in an oven, which can enhance the rate of desorption.

### Mechanism

**Figure 7:**
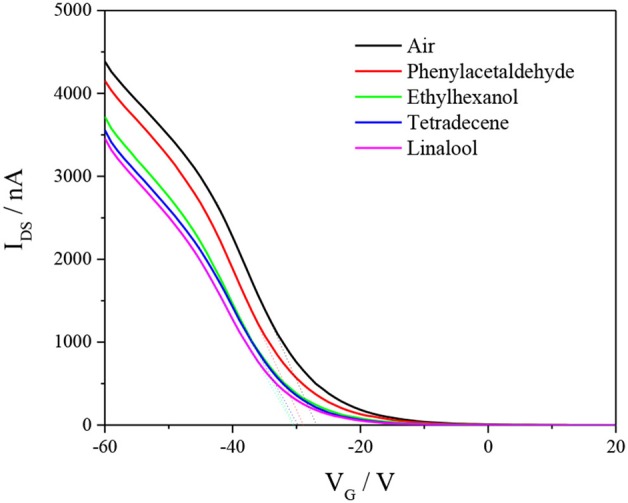
Transfer characteristics (I_DS_-V_G_ curves at V_DS_ = −0.1 V) of CuTPP-SWNTs in the presence of air and different saturated VOCs.

The sensing mechanism for the field-effect transistor includes four aspects: electrostatic gating, changes in gate coupling, Schottky barrier effects, and carrier mobility changes (Heller et al., 2008). CuTPP-SWNTs were selected to investigate the sensing mechanism upon interaction with organic gas molecules. Prior to FET detection, CuTPP-SWNTs were exposed to steaming air or saturated VOC vapors for 60 min to ensure that the gas molecules could interact adequately with the gas-sensitive material of CuTPP-SWNTs. Figure 7 shows the transfer characteristics (I_DS_-V_G_) curves. Compared to the FET curve in dry air, the I_DS_-V_G_ curves of phenylacetaldehyde shifted in the negative direction, and the threshold gate voltage (V_TH_) was decreased, which was mainly ascribed to the electrostatic gating effect. According to the mobility equation, the values of carrier mobility of ethyl hexanol, tetradecane, and linalool reduced compared to the mobility for dry air. Thus, a threshold voltage shift and change in carrier mobility in the case of exposure of the CuTPP-SWNT hybrid to ethyl hexanol, tetradecane, and linalool indicate that the sensing mechanism is mainly governed by electrostatic gating and carrier mobility changes in combination.

### Mathematical Model for VOC Analysis

Pattern recognition tools can provide powerful multivariate analysis methods to extract specific non-related information from complex correlations and have been widely applied in the multisensory field. Herein, we chose linear and non-linear models to process the data collected by the hybrid chemiresistor sensor arrays for the qualitative and quantitative analysis of the four VOCs. Principal component analysis (PCA) was performed, and the results are shown in Figure S5 and Table S6.

#### Partial Least Square Regression (PLSR)

PLSR (Mehmood et al., 2012), a multivariate calibration model, was used to deal with the two multivariable matrices (X0 and Y0) with significantly redundant variations. It is realized by extracting predictor variables (X and Y) from the block X0-matrix and the block Y0-matrix and then analyzing and establishing the relationship between the block X-matrix and the block Y-matrix. PLSR combines the merits of principal component analysis and multiple linear regression. This analysis also suits small samples with a simplified data structure.

**Figure 8:**
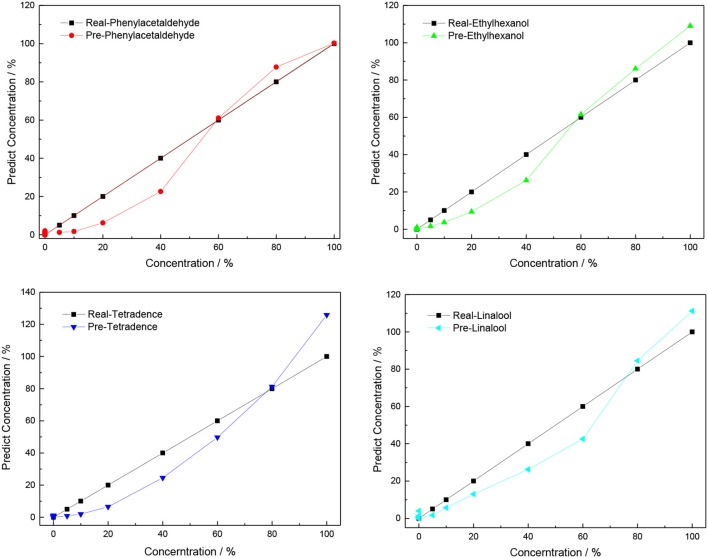
Predicted concentration against true concentration of four VOCs for the PLS model.

For this experiment, the X0-matrix consists of 28 columns and 7 rows, with different concentrations (corresponding to 5, 10, 20, 40, 60, 80, and 100% saturated vapors) of four VOCs as the columns and normalized responses of MP-functionalized SWNTs (bare SWNTs, TPP-SWNTs, CuTPP-SWNTs, FeTPP-SWNTs, ZnTPP-SWNTs, CuOEP-SWNTs, MnOEP-SWNTs) as rows, and the Y0-matrix consists of 28 columns and 4 rows by using the real concentration as the columns and the four VOCs as the rows. As the normalized responses (X_0_-matrix) are non-linear with the concentrations of VOCs (Y_0_-matrix), these data matrices need to be pre-processed prior to use: X = X_0_, Y = Sqrt (Y_0_). After the pre-treatment, the relationship between X and Y approximates linearity. The X-matrix and Y-matrix are inputted into the PLSR model, and the result is shown in Figure S6 and Figure 8. We found that the correlation coefficient between the real concentration and fit concentration was much higher and the standard deviation was lower than the matrix without pretreatment; the data are presented.

#### Artificial Neural Network

**Figure 9:**
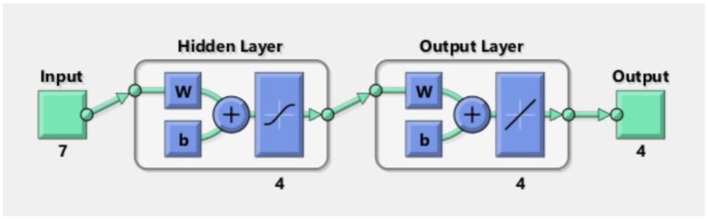
Type of architecture of the neural network.

**Figure 10:**
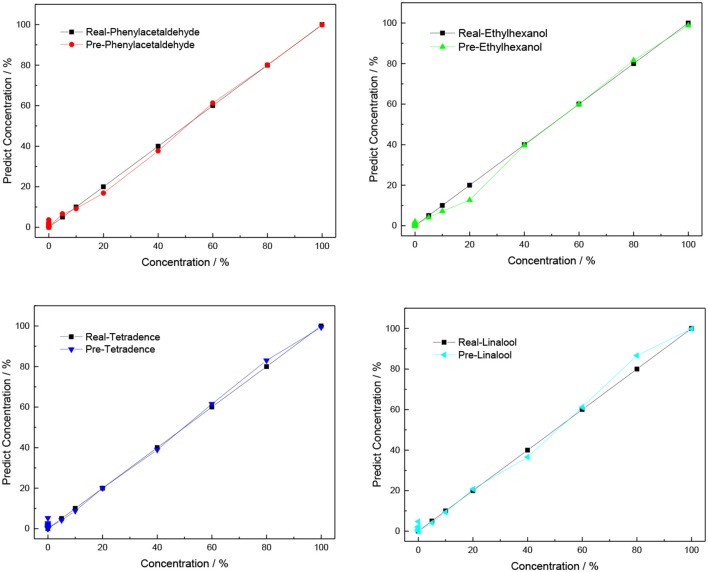
Predicted concentration against true concentration of four VOCs for the ANN model.

An artificial neural network (ANN) (Asilturk and Cunkas, 2011) is a flexible mathematical structure that is capable of identifying complex non-linear relationships between input and output data sets. ANN models have been found to be useful and efficient, particularly in problems for which the characteristics of the processes are difficult to describe using physical equations. The overall structure is shown in Figure 9. The procedures of ANN are divided into three main parts: an input layer, hidden layers, and an output layer. In this model, the X matrix is the input layer, which consists of 28 columns and 7 rows filled by using different concentrations (corresponding to 5, 10, 20, 40, 60, 80, and 100% saturated vapors) of four VOCs as the columns and normalized responses of MP-functionalized SWNTs (bare SWNTs, TPP-SWNTs, CuTPP-SWNTs, FeTPP-SWNTs, ZnTPP-SWNTs, CuOEP-SWNTs, and MnOEP-SWNTs) as rows. The Y matrix is selected as an output layer, which consists of 4 columns and 28 rows, with the four concentrations in each row corresponding to each column of X. The data of the X matrix are divided randomly into three sets: 60% as training samples, 10% as validation samples, and 30% as testing samples. During learning, output values from the ANN are compared to true values, and the coupling weights are adjusted to give a minimum sum of square errors. After testing and comparing, we found that four nodes of the hidden layer can cause the average relative error to reach a minimum, shown in Figure S7 and Figure 10.

Among the three mathematical models, PCA is used only for qualitative analysis of VOCs emitted by infected citrus trees, and it is hard to acquire accurate results when the gas concentration of any VOC is below 20%. Both PLSR and ANN are simple methods that can offer qualitative and quantitative information, which is widely employed in stoichiometry. The correlation coefficient (*R*_0_) and root mean square error (R_SMT_) between real concentrations and predicted concentrations are shown in Table 1. We found that the best result is achieved with the utilization of the ANN model because the values of R_0_ for these VOCs are higher and the R_SMT_ is lower than with PLS and SPLS. This demonstrates that a non-linear regression model is well-suited for non-linear data processing.

**Table 1:** The correlation coefficient (R_0_) and root mean square error (R_SMT_) between real concentrations and predicted concentrations.

	**Phenylacetaldehyde**		**Ethylhexanol**		**Tetradecene**		**Linalool**	
	**R_**0**_**	**R_**SMT**_**	**R_**0**_**	**R_**SMT**_**	**R_**0**_**	**R_**SMT**_**	**R_**0**_**	**R_**SMT**_**
PLSR	0.92816	9.58762	0.98718	4.11176	0.98303	4.72537	0.9762	5.58669
SPLS	0.98321	4.82493	0.98924	4.12993	0.9734	6.75741	0.98067	5.14909
ANN	0.99883	1.33108	0.99784	1.69709	0.99861	1.53506	0.99794	1.80906

## Conclusions

Volatile organic compounds (VOCs; phenylacetaldehyde, tetradecene, linalool, and ethylhexanol) released by infected citrus trees are associated with plant metabolism and can serve as biomarkers for the detection of Huanglongbing disease (HLB). In conclusion, we have fabricated hybrid chemiresistor sensor arrays based on SWNTs functionalized with different MPs to determine changes in the concentrations of four VOCs emitted by infected citrus trees, which are used to diagnose HLB at an asymptomatic stage. Optical and electrochemical approaches have been applied to examine the electrical characterization of SWNTs before and after functionalization with MPs and have shown that MPs can attach to SWNTs to form a stable membrane. MP-SWNTs improved the sensitivity to the various VOCs tested, and are shown to have differences in sensing performance. To obtain accurate concentrations, the test data collected by the hybrid chemiresistor sensor arrays were analyzed using the PCA, PLSR, and ANN techniques. By comparing these three results, it was found that ANN was much better suited to the non-linear data processing required.

## Data Availability Statement

The raw data supporting the conclusions of this article will be made available by the authors, without undue reservation, to any qualified researcher.

## Author Contributions

HW: data curation, formal analysis, methodology, software, writing—original draft, reviewing, and editing. PR, TP, CV, and XY: formal analysis and methodology. GL: supervision, reviewing, and editing. AM: conceptualization, funding, resources, supervision, methodology, reviewing, and editing.

## Conflict of Interest

The authors declare that the research was conducted in the absence of any commercial or financial relationships that could be construed as a potential conflict of interest.
