# Supplementary material for: Asymptomatic Diagnosis of Huanglongbing Disease Using Metalloporphyrin Functionalized Single-Walled Carbon Nanotubes Sensor Arrays
Source: Front Chem. 2020 May 12;8:362. doi: 10.3389/fchem.2020.00362 (PMC7237200; doi:10.3389/fchem.2020.00362)
Supplement: Supplementary file 1 [file Data_Sheet_1.docx]

Asymptomatic Diagnosis of Huanglongbing Disease using Metalloporphyrin Functionalized Single-Walled Carbon Nanotubes Sensor Arrays

*Hui Wang ^1,2,3,5^, Pankaj Ramnani ^3^, Tung Pham ^3^, Claudia Chaves Villarreal^,4^, Xuejun Yu ^3^, Gang Liu ^1^* and Ashok Mulchandani ^3^**

*^1^ Key Laboratory of Modern Precision Agriculture System Integration Research, Ministry of Education and Key Laboratory of Agricultural Information Acquisition Technology, Ministry of Agriculture China Agricultural University, Beijing 100083, P.R. China；**^2^ State Key Laboratory of Animal Nutrition, Institute of Animal Science, Chinese Academy of Agricultural Sciences, Beijing, 100193, China; ^3^ Department of Chemical and Environmental Engineering and Material Science and Engineering Program,, University of California-Riverside, Riverside, California 92521, United States; ^4^ Escuela de Ciencia e Ingeniería de Materiales, Centro de Investigación y Extensión de Materiales, Instituto Tecnológico de Costa Rica, Cartago 30101 Costa Rica ^5^ Research Institute of Wood Industry, Chinese Academy of Forestry, Beijing 100091, China.*


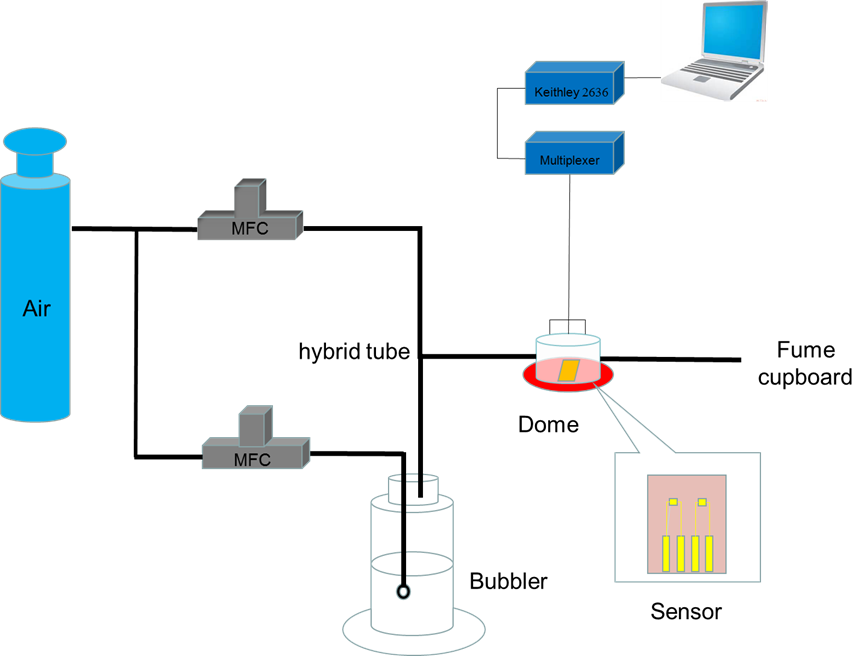


**Figure S1.** Schematic representation of the gas sensing system

**Figure S2.** The resistance of bare SWNTs and CuTPP-SWNTs at different voltages.

**Figure S3.** The real-time relative responses of bare SWNTs towards different concentrations of four VOCs varying from 5% to 100%.

**Figure S4.** The real-time relative responses of bare SWNTs and CuTPP-SWNT towards different concentrations of tetradecene varying from 5% to 100%.

**Table S1.** **The relative response of seven MPs-SWNTs for different concentrations of phenylacetaldehyde.**

| Satuarted vapors of phenylacetaldehyde | Bare SWNTs  （%） | TPP-  SWNTs  （%） | ZnTPP-SWNTs  （%） | FeTPP-SWNTs  （%） | CuTPP-SWNTs  （%） | CuOEP-SWNTs  （%） | MnOEP-SWNTs  （%） |
| --- | --- | --- | --- | --- | --- | --- | --- |
| 5% | 0.23±0.20 | 1.31±0.98 | 0.50±0.19 | -0.50±0.10 | -0.07±0.05 | 0.00±0.00 | -0.21±0.12 |
| 10% | 0.48±0.32 | 2.81±2.04 | 0.84±0.31 | -0.55±0.06 | -0.23±0.14 | -0.49±0.39 | -0.40±0.18 |
| 20% | 0.69±0.21 | 8.29±2.68 | 2.17±0.22 | -0.81±0.42 | -4.68±3.71 | -1.15±0.31 | -1.80±0.11 |
| 40% | 1.55±0.54 | 23.74±5.45 | 6.53±0.71 | -2.00±0.55 | -5.84±4.69 | -1.87±0.37 | -6.25±1.49 |
| 60% | 3.62±1.37 | 56.27±7.64 | 9.76±1.31 | -4.01±0.79 | -8.61±5.81 | -2.59±0.39 | -11.26±1.96 |
| 80% | 5.72±1.54 | 87.68±14.06 | 13.20±1.79 | -4.57±0.76 | -12.36±6.90 | -4.812±0.43 | -14.99±2.26 |
| 100% | 8.29±2.54 | 155.33±23.75 | 16.97±2.21 | -4.60±0.64 | -14.22±7.46 | -7.35±0.74 | -16.63±2.16 |

**Table S2. The relative response of seven MPs-SWNTs for different concentrations of ethylhexanol**

| Satuarted vapors of ethylhexanol | Bare SWNTs  （%） | TPP-SWNTs  （%） | ZnTPP-SWNTs  （%） | FeTPP-SWNTs  （%） | CuTPP-SWNTs  （%） | CuOEP-SWNTs  （%） | MnOEP-SWNTs  （%） |
| --- | --- | --- | --- | --- | --- | --- | --- |
| 5% | -0.09±0.03 | 0.38±0.14 | 0.03±0.01 | -0.01±0.01 | -0.02±0.02 | -0.89±0.44 | 0.00±0.00 |
| 10% | -0.36±0.37 | 0.85±0.30 | 0.97±0.08 | -0.06±0.04 | -1.50±0.55 | -3.22±0.63 | -0.39±0.48 |
| 20% | -0.64±0.09 | 1.43±0.21 | 2.75±1.35 | -0.19±0.11 | -2.00±0.81 | -6.33±1.45 | -1.49±0.81 |
| 40% | -5.01±1.31 | 4.00±0.32 | 9.59±5.62 | -0.37±0.17 | -8.89±2.19 | -22.92±5.96 | -9.19±2.82 |
| 60% | -7.95±2.06 | 9.08±1.59 | 13.63±4.69 | -0.56±0.27 | -19.90±3.91 | -36.82±8.63 | -20.32±5.02 |
| 80% | -9.01±2.29 | 18.98±2.93 | 18.89±6.67 | -0.76±0.36 | -28.69±5.41 | -44.03±9.42 | -25.55±6.82 |
| 100% | -9.26±2.29 | 39.46±3.43 | 23.53±7.94 | -0.65±0.24 | -35.57±5.71 | -48.92±9.42 | -29.27±7.82 |

**Table 3. The relative response of seven MPs-SWNTs for different concentrations of tetradecene**

| Satuarted vapors of tetradecene | Bare SWNTs  （%） | TPP-SWNTs  （%） | ZnTPP-SWNTs  （%） | FeTPP-SWNTs  （%） | CuTPP-SWNTs  （%） | CuOEP-SWNTs  （%） | MnOEP-SWNTs  （%） |
| --- | --- | --- | --- | --- | --- | --- | --- |
| 5% | 0.23±0.23 | 0.55±0.17 | 0.63±0.15 | 0.23±0.15 | -0.57±0.65 | -0.28±0.10 | -0.27±0.13 |
| 10% | 0.24±0.19 | 1.13±0.36 | 1.07±0.19 | 0.41±0.09 | -3.40±1.30 | -0.86±0.27 | -0.84±0.46 |
| 20% | 0.26±0.16 | 1.73±0.54 | 1.36±0.55 | 0.98±0.12 | -9.18±1.90 | -1.37±0.41 | -1.86±0.60 |
| 40% | 0.70±0.21 | 3.43±0.65 | 2.83±0.47 | 2.56±0.44 | -15.15±2.82 | -1.72±0.51 | -7.44±1.93 |
| 60% | 0.79±0.27 | 7.39±1.37 | 3.54±1.04 | 4.61±0.92 | -22.07±3.72 | -1.83±0.69 | -9.80±3.45 |
| 80% | 0.86±0.28 | 14.88±4.32 | 4.02±1.08 | 6.44±1.46 | -30.61±4.38 | -3.09±0.84 | -10.46±3.54 |
| 100% | 0.83±0.32 | 25.80±7.37 | 4.04±1.09 | 9.09±2.33 | -37.25±5.01 | -4.84±0.92 | -11.23±3.34 |

**Table 4. The relative response of seven MPs-SWNTs for different concentrations of linalool**

| Satuarted vapors of linalool | Bare SWNTs  （%） | TPP-SWNTs  （%） | ZnTPP-SWNTs  （%） | FeTPP-SWNTs  （%） | CuTPP-SWNTs  （%） | CuOEP-SWNTs  （%） | MnOEP-SWNTs  （%） |
| --- | --- | --- | --- | --- | --- | --- | --- |
| 5% | 0.25±0.09 | 1.48±0.21 | 0.45±0.32 | 0.04±0.04 | 0.04±0.02 | 0.08±0.17 | -0.06±0.07 |
| 10% | 0.20±0.27 | 2.09±0.51 | 0.99±0.75 | 0.13±0.03 | 0.10±0.05 | 0.50±0.11 | -0.42±0.17 |
| 20% | 0.33±0.20 | 3.16±0.63 | 1.67±1.13 | 0.60±0.17 | 2.21±0.89 | 1.07±0.31 | -0.56±0.79 |
| 40% | 1.00±0.27 | 6.15±0.63 | 3.34±1.61 | 0.97±0.29 | 4.98±0.69 | 2.16±0.87 | -2.02±0.46 |
| 60% | 2.50±1.20 | 13.15±3.04 | 5.65±2.13 | 1.72±0.53 | 12.38±1.82 | 3.95±1.41 | -2.95±0.53 |
| 80% | 4.32±2.09 | 23.46±5.57 | 8.94±3.08 | 2.68±0.82 | 22.24±2.53 | 6.65±2.34 | -3.20±0.46 |
| 100% | 7.23±4.01 | 38.37±10.54 | 12.72±4.24 | 4.292±1.25 | 31.88±3.4 | 9.89±3.16 | -3.40±0.42 |

**Table S5. The lowest detection limits of four VOCs.**

| Different VOC | Gas senor | Vapor Pressure | Limit of Detection |
| --- | --- | --- | --- |
| Phenylacetaldehyde | TPP-SWWNTs | 0.36 mmHg | 4.86 ppm |
| Ethylhexanol | CuOEP-SWNTs | 0.368 mmHg | 10.15 ppm |
| Tetradecene | ZnTPP-SWNTs | 0.0905 mmHg | 0.18ppm |
| Linalool | TPP-SWNTs | 0.01 mmHg | 0.61ppm |

### Principal component analysis (PCA)

PCA is a multivariate statistical method through sophisticated underlying mathematical principles to extract a few of independent variables called principal components from an large number of possibly dependent variables without losing too much information. Brief, it uses an orthogonal transformation to convert a high-order matrices into a low-order matrices, which has widely used for qualitative analysis in the gas sensing filed. In our experiment, the higher data matrix consists of 7 columns and 28 rows by using normalized responses of MPs functionalized SWNTs (bare SWNTs, TPP-SWNTs, CuTPP-SWNTs, FeTPP-SWNTs, ZnTPP-SWNTs, CuOEP-SWNTs, MnOEP-SWNTs) as the columns and different concentrations (corresponding to 5%, 10%, 20%, 40%, 60%, 80% and 100% saturated vapors) of four VOCs as the rows, which is used as the independent variable to principal component analysis. The PCA scores of four VOCs are shown in Fig. S5.

The first three principal components in Table S5 are 51.99%, 26.83% and 13.01% of the total variance respectively and contains 91.82% of the total variance, which can represent the high-order matrices of the hybrid chemiresistor arrays. The four VOCs are presented by different colors shown in Fig. S5(A). It can be observed that the plots of different colors are clustered in different direction and separated clearly above 20% saturated vapors, while there are closed and overlap among the plots that are hard for the gas identity. The reason ascribed to the poor sensitivity and selectivity of MPs-SWNTs at the lower concentration.

(A) (B)

**Figure S5.** PCA score plots using seven sensors (A) the VOCs concentration ranging from 5 % to 100 %, (B) the VOCs concentration ranging from 20 % to 100 %

**Table S6.** Eigenvalues of the Correlation Matrix

|  | Eigenvalue | Percentage of Variance | Cumulative |
| --- | --- | --- | --- |
| 1 | 3.63908 | 51.99% | 51.99% |
| 2 | 1.87794 | 26.83% | 78.81% |
| 3 | 0.91066 | 13.01% | 91.82% |
| 4 | 0.50856 | 7.27% | 99.09% |
| 5 | 0.054 | 0.77% | 99.86% |
| 6 | 0.00771 | 0.11% | 99.97% |
| 7 | 0.00204 | 0.03% | 100.00% |

**Figure S6.** PLS score plots.

**Figure S7.** ANN score plots.
